# Underutilisation of GeneXpert devices for TB diagnosis: a missed opportunity

**DOI:** 10.5588/ijtldopen.25.0143

**Published:** 2025-07-09

**Authors:** J. Dörfler, O.G. Ravololohanitra, T. Decroo, M.A. Franke, J. Emmrich, G.I. Pasteur, E.A. Harizaka, N. Muller

**Affiliations:** ^1^ Department of Infectious Diseases and Critical Care Medicine, Charité – Universitätsmedizin Berlin, corporate member of Freie Universität Berlin and Humboldt-Universität zu Berlin, Berlin, Germany;; ^2^ Institute of Tropical Medicine, Antwerp, Belgium;; ^3^ Doctors for Madagascar, Antananarivo, Madagascar;; ^4^ Charité Center for Global Health, Charité – Universitätsmedizin Berlin, corporate member of Freie Universität Berlin and Humboldt-Universität zu Berlin, Berlin, Germany;; ^5^ Ärzte für Madagaskar e.V., Leipzig, Germany;; ^6^ Berlin Institute of Health, Berlin, Germany.

**Keywords:** tuberculosis, Madagascar, molecular diagnostics, capacity building

Dear Editor,

The COVID-19 pandemic spurred international donations of diagnostic equipment, including GeneXpert (GX) technology (Cepheid, Inc), to strengthen diagnostic capacity in decentralized, resource-constrained settings.^1^ Through such support, Madagascar, a high-TB incidence country (with an estimated 233 cases per 100,000 in 2023)^2^ expanded and decentralized molecular testing. In 2022, Madagascar received 10 GX devices, with an additional 50 donated during the COVID-19 pandemic. However, despite alignment with WHO recommendations to use GX as a first-line test for all TB cases, smear microscopy remains the predominant diagnostic method.^2^ Molecular testing was used in only 15% of new drug-sensitive TB cases in 2023,^2^ and the overall diagnostic gap remains large, with only 65% of 63,000 estimated TB cases reported in 2023.^3^

We conducted a cross-sectional investigation as part of a mixed-methods study to examine laboratory infrastructure, GX device readiness and usage across three administrative regions in Madagascar, to assess the potential to improve TB diagnosis. The study area (Atsimo-Andrefana, Androy, and Anosy), has a predominantly rural population of 4 million,^4^ and was chosen due to its above-average TB notification rate (310/100,000, vs 149/100,000 nationally, 2022), with low levels of bacteriological confirmation (54%, 2022).^5^ The diagnostic pathway for TB in Madagascar involves active case finding by community health workers (CHWs), who refer patients to diagnostic and treatment centers (CDTs; 1/100,000 population).^2^ CDTs provide free TB testing, perform diagnostics for treatment initiation and treatment monitoring, and ensure collection and referral of clinical samples to regional referral laboratories for further analysis, if needed. All laboratories operate under the supervision of a National Reference Laboratory. The available GX devices were distributed by the Malagasy Ministry of Health and are accessible for diagnosing various infectious diseases, including TB.^2^ Using total population sampling, we included all TB referral laboratories (n=7) with ≥1 GX device available in 2024. All were public urban facilities, serving as referral centers for surrounding rural populations. Data were collected during site visits from June–August 2024 using a context-adapted assessment tool based on WHO’s Service availability and readiness assessment.^6^ We reviewed laboratory infrastructure, equipment, GX test kits, consumables, and operational standards, including diagnostic manuals, calibration schedules and maintenance trained staff. We assessed GX diagnostic workload by recording MTB/RIF Ultra tests performed in the previous 3 months, comparing actual usage with theoretical capacity.^7^ Data were analyzed using JMP® Pro 17.1.0. Ethics approval and consent to participate: Ethics approval for this study was granted by the Comité d’Évaluation Éthique pour la Recherche Biomédicale (Ethics Review Committee for Biomedical Research), Antananarivo, Madagascar on May 6th, 2024 (number 005/2024 – CEERINSPC/IRB) and the Institute of Tropical Medicine, Antwerp, Belgium on February 14th 2024 (number 1742/24). Informed consent was waived by the ethics committee as the assessment was solely based on health facility data and did not include human participant data. Formal permission for data collection and analysis was additionally sought and received from the regional public health authorities and the regional program managers for infectious diseases in the three study regions.

Across 7 laboratories, 13 GX devices were available (see Figure); 1 was donated before 2022 and 12 were donated in 2022/2023.^2^ In total, 7 devices (54%) were never installed – 5 (38%) awaited transfer to other laboratories, 2 (15%) remained uninstalled due to infrastructure and administrative barriers. Of 6 installed devices (46%), 3 (23%) were actively used for TB diagnosis, 1 (8%) for HIV diagnosis, and 2 installed devices (15%) remained unused due to lack of air conditioning or administrative barriers. A total of 2,080 Xpert MTB/RIF Ultra cartridges were available in 5/7 (71.4%) laboratories (median 400/lab (IQR: 190–650)). Cartridge distribution varied by region: Atsimo-Andrefana (TB notification rate: 415/100,000) had 1,200 cartridges, Androy (219/100,000) had 800, and Anosy (180/100,000) had only 80. Details from the assessment of infrastructure, equipment, consumables, and operational standards are shown in the Table. From March–May 2024, three active GXs (4-, 8-, and 16-module) performed 1,022 MTB/RIF Ultra tests, averaging 340 tests per month. Given a theoretical test volume of 2,800 tests per month,^7^ this represents utilisation of just 12% of the theoretical capacity. None of the laboratories performed mycobacterial culture.

**Figure.**
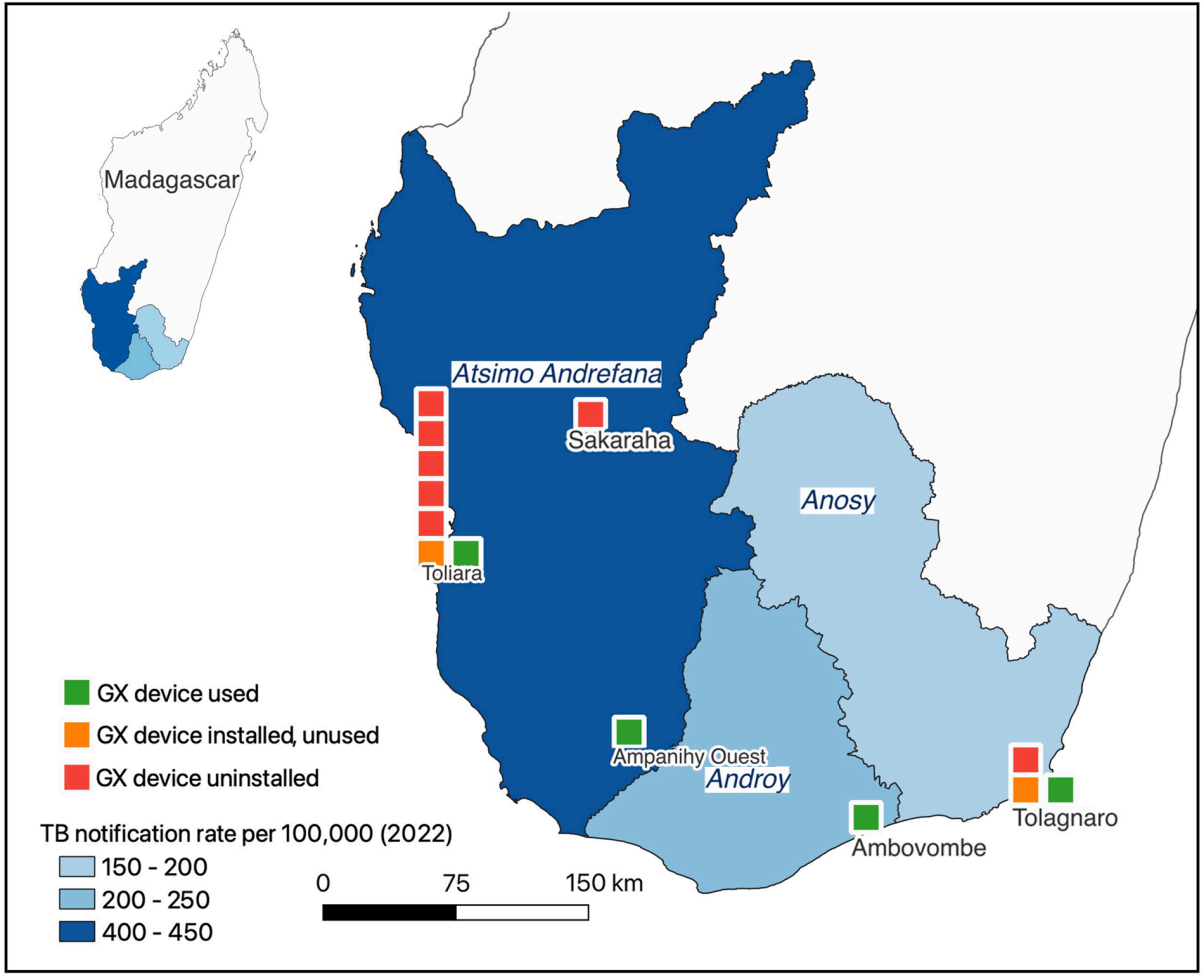
Map showing local TB notification rates, the availability and utilization status of GeneXpert devices across TB referral laboratories in southern Madagascar, 2024.

**Table.** The infrastructure, equipment, consumables and availability of operational standards at all referral laboratories (n=7), labs with active GeneXpert (GX) use (n=4), and labs with unused GXs (n=3).

	Total labs (n=7)	Labs with used GXs (n=4)	Labs with unused GXs (n=3)
	n (%)	n (%)	n (%)
**Infrastructure**			
Dedicated GX room	6 (86)	3 (75)	3 (100)
Stable electricity supply GX	3 (43)	2 (50)	1 (33)
A/C unit available	*5 (71)*	3 (75)	*2 (67)*
*Functioning A/C*	*3 (43)*	*3 (75)*	*0 (0)*
Continuous water supply	3 (43)	2 (50)	1 (33)
Wastewater disposal system	6/6 (100)	3/3 (100)	3 (100)
Incinerator	5 (71)	2 (50)	3 (100)
**Equipment**			
Functioning refrigerator	7 (100)	4 (100)	3 (100)
Functioning microscope	6 (86)	4 (100)	2 (67)
Functioning centrifuge	3 (43)	0 (0)	3 (100)
**Consumables**			
PPE gloves	5 (71)	3 (75)	2 (67)
Sample storage capacity	6 (86)	3 (75)	3 (100)
Cartridge storage capacity	6 (86)	4 (100)	2 (67)
Sufficient cartridge stock	5 (71)	3 (75)	2 (67)
MTB/RIF Ultra cartridges (n)	2,080	1,700	380
**Operational standards**			
GX TB diagnostic manual	4 (57)	3 (75)	1 (33)
Trained staff for maintenance	4 (57)	2 (50)	2 (67)
Maintenance calibration schedule	3 (43)	2 (50)	1 (33)
Regular GX calibration	3 (43)	2 (50)	1 (33)

A/C = air conditioning; PPE = personal protective equipment;

Our assessment highlights a significant gap between GX availability and its actual usage in southern Madagascar, with less than one-third of GX devices installed and used. The identified infrastructural gaps align with findings from other high TB burden countries,^1,8,9^ underscoring the disconnect between GX donations and the necessary infrastructure upgrades for their effective use.^10,11^ Although none of GX devices were nonfunctional due to inadequate operational standards, gaps existed in routine calibration, protocol adherence and trained maintenance staff. This highlights the substantial underuse of available GX diagnostic capacity, likely driven by infrastructure barriers (such as instable electricity supply) and low testing demand. Healthcare access barriers,^12^ and insufficient active case finding by CHWs (despite their inclusion in the NTP’s National Strategic Plan)^2,13^ result in late-stage disease presentation and gaps in the TB care cascade. For drug-sensitive TB, losses of 13% at CDT access and 26% at diagnosis have been documented nationally.^13^ The high GX test positivity rate of 23.5%, alongside Madagascar’s consistently high smear microscopy positivity rates (25% during 2014–2021),^2^ underscores systematic gaps in case detection and suggest a targeted testing approach of populations with a high pre-test probability of TB, rather than broad population screening of all presumptive cases.^2^

Underutilization of available GX capacity is a common problem. Nationally, only 28% of annual GX testing capacity was used in 2021,^2^ whereas global GX utilization rates range from 20–74%.^1,14^ A range of factors lead to GX underutilisation, including the lack of diagnostic guidelines, trained staff, sample transport challenges, cartridge stockouts and equipment failure.^1,9,15^ In our setting, misalignment between cartridge availability and local TB notification rates likely further impacted GX underutilisation, highlighting the need for epidemiologically-informed distribution strategies. Investigating and addressing specific barriers to GX service accessibility and demand is essential to enhance diagnostic impact and improve timely, accurate TB testing in Madagascar.

A limitation to our cross-sectional design is that it captures only a snapshot of laboratory capacity without temporal patterns or causality. Longitudinal studies are needed to assess the evolution of diagnostic capacity. Additionally, our focus on southern Madagascar limits generalizability to other regions. Investigation of GX device availability and functionality across the country would improve understanding of regional variations, aligning with the NTP’s recommendation for a comprehensive review of device positioning, infrastructure, supply and staffing.^2^

To conclude, sustained, active collaboration among funders, donors and recipients is essential to ensure that donated equipment is used effectively to expand and decentralize TB diagnostics. Realising the full potential of GX technology and maintaining functionality requires investment in laboratory infrastructure, training, operating and maintenance guidance, reliable service contracts and supportive follow up.^9^ Locally experienced partners, such as civil society organizations, can help to facilitate effective partnerships fostering sustainable GX implementation.^10^
